# Interpretation of Quantum Theory: The Quantum “Grue-Bleen” Problem

**DOI:** 10.3390/e24091268

**Published:** 2022-09-09

**Authors:** Benjamin Schumacher, Michael D. Westmoreland

**Affiliations:** 1 Department of Physics, Kenyon College, Gambier, OH 43022, USA; 2 Department of Mathematics, Denison University, Granville, OH 43023, USA

**Keywords:** interpretation of quantum mechanics, quantum foundations, many-worlds interpretation

## Abstract

We present a critique of the many-world interpretation of quantum mechanics, based on different “pictures” that describe the time evolution of an isolated quantum system. Without an externally imposed frame to restrict these possible pictures, the theory cannot yield non-trivial interpretational statements. This is analogous to Goodman’s famous “grue-bleen” problem of language and induction. Using a general framework applicable to many kinds of dynamical theories, we try to identify the kind of additional structure (if any) required for the meaningful interpretation of a theory. We find that the “grue-bleen” problem is not restricted to quantum mechanics, but also affects other theories including classical Hamiltonian mechanics. For all such theories, absent external frame information, an isolated system has no interpretation.

## 1. Introduction

### 1.1. The Many-Worlds Interpretation

Any critique of the many-worlds interpretation of quantum mechanics ought to begin by praising it. In the simplest form of the interpretation, such as that presented by Everett in 1957 [1,2], the universe is regarded as a closed quantum system. Its state vector (Everett’s “universal wave function”) evolves unitarily according to an internal Hamiltonian. Measurements and the emergence of classical phenomena are described entirely by this evolution. “Observables” are simply dynamical variables described by operators. No separate “measurement process” or “wave function collapse” ideas are invoked.

Thus, consider a laboratory measurement of $S_{z}$ on a spin-1/2 particle. This is nothing more than an interaction among the particle, the lab apparatus, and the conscious observer, all of which are subsystems of the overall quantum universe. Initially, the particle is in the state $|\psi_{0}\rangle =\alpha |↑\rangle +\beta |↓\rangle$. The apparatus and the observer are in initial states $|0\rangle$ and $|“\mathrm{ready}”\rangle$, respectively. Now the particle and the apparatus interact and become correlated:

(1) $$|\psi_{0}\rangle ⊗|0\rangle ⊗|“\mathrm{ready}”\rangle ⟶\left(\alpha |↑\rangle ⊗|+\frac{\hbar }{2}\rangle +\beta |↓\rangle ⊗|-\frac{\hbar }{2}\rangle \right)⊗|“\mathrm{ready}”\rangle ,$$

where $|+\frac{\hbar }{2}\rangle$ and $|-\frac{\hbar }{2}\rangle$ are apparatus states representing the two possible measurement results. The observer next interacts with the apparatus by reading its output, leading to a final state

(2) $$\alpha |↑\rangle ⊗|+\frac{\hbar }{2}\rangle ⊗|“\mathrm{up}”\rangle +\beta |↓\rangle ⊗|-\frac{\hbar }{2}\rangle ⊗|“\mathrm{down}”\rangle .$$

The memory record of the observer (“up” or “down”) has become correlated to both the original spin and the reading on the apparatus. The two components of the superposition in Equation (2) are called “branches” or “worlds”. Since all subsequent evolution of the system is linear, the branches effectively evolve independently. The observer can condition predictions of the future behavior of the particle on his own memory record—for example, if his memory reads “spin up”, then he may regard the state of the spin as $|↑\rangle$. No collapse has occurred; both measurement outcomes are still present in the overall state. However, conditioning on a particular memory record yields a *relative state* of the particle that corresponds to that record. In the same way, if other observers read the apparatus or perform independent measurements of the same observable, all observers will find that their memory records are consistent.

Here is another way to look at this process. Consider the dynamical variable C on the spin–observer subsystem given by:

(3) $$C=|↑\rangle \;\langle ↑|⊗|“\mathrm{up}”\rangle \;\langle “\mathrm{up}”|+|↓\rangle \;\langle ↓|⊗|“\mathrm{down}”\rangle \;\langle “\mathrm{down}”|.$$

This variable is a projection onto the subspace of system states in which the spin state and the observer memory state agree. At the start of the measurement process, the “expectation” $\langle C\rangle =\langle \Psi |C|\Psi \rangle =0$, but at the end $\langle C\rangle =1$. The evolution of $\langle C\rangle$ tells us that a correlation has emerged between the spin and the memory record. Note that this does not depend on a probabilistic interpretation of the expectation $\langle C\rangle$. The expectation $\langle C\rangle$ simply indicates the relationship between the system state and eigenstates of C that are either uncorrelated ($\langle C\rangle =0$) or correlated ($\langle C\rangle =1$).

There are many things to like about the many-worlds account. It entails no processes other than the usual dynamical evolution according to the Schrödinger equation. It explains at least some characteristics of a measurement, such as the repeatability and consistency of the observers’ records. It focuses attention on the actual physical interactions involved in the measurement process. Some details may be tricky, such as the identification of ${\left|\alpha \right|}^{2}$ and ${\left|\beta \right|}^{2}$ as observed outcome probabilities in repeated measurements [3,4]. Nevertheless, the many-worlds idea has proven to be very fruitful, for example, in motivating the analysis of decoherence processes [5] and their role in the emergence of quasi-classical behavior in quantum systems [6,7].

The essential idea of the many-worlds program was formulated by Bryce DeWitt [8] in the following maxim:

The mathematical formalism of the quantum theory is capable of yielding its own interpretation.

DeWitt called this the “EWG metatheorem”, after Everett and two other early exponents of the interpretation, John Wheeler [9] and Neill Graham [10]. DeWitt’s claim is that the only necessary foundations for sensible interpretational statements about quantum theory are already present in the mathematics of the Hilbert space of states and the time evolution of the global system. Nothing outside of the system and its unitary evolution is required.

### 1.2. Two Universes, Two Pictures

Consider a closed quantum “universe”, which we will call Q. System Q is composite with many subsystems. Its time evolution is unitary, so that the state at any give time is

(4) $$|\Psi (t)\rangle =U\left(t\right)|\Psi_{0}\rangle$$

for evolution operator U(t) and initial state $|\Psi_{0}\rangle$. For convenience, we will refer to this as the “actual” time evolution of the system.

To make our mathematical discussion straightforward, we imagine that Q is bounded in space, so that its Hilbert space $H^{\left(Q\right)}$ has a discrete countable basis set. (The Hamiltonian eigenbasis would be an example of such.) If we further impose an upper limit $E_{\max}$ to the allowed energy of the system, the resulting $H^{\left(Q\right)}$ is finite-dimensional. Note that this scarcely limits the possible complexity of Q. The system may still contain a multitude of subsystems with complicated behavior. The subsystems may exchange information and energy. Some of the subsystems may function as “observers”, interacting with their surroundings and recording data in their internal memory states.

According to the DeWitt maxim, the initial state $|\Psi_{0}\rangle$ and time evolution operator U(t) suffice to specify a many-worlds interpretation of what happens in Q. One way to describe this is to consider a large collection of dynamical variables $A_{1}$, $A_{2}$, etc. These may represent particle positions, observer memory states, correlation functions, and so on. From the time-dependent expectations ${\langle A_{k}\rangle }_{t}$, we identify processes such as measurements, decoherence, and communication. (Indeed, if the set $\left\{A_{k}\right\}$ is large enough, we can completely reconstruct the time evolution $|\Psi (t)\rangle$ from the expectations ${\langle A_{k}\rangle }_{t}$.) We can in principle tell what the system “looks like” to various observer subsystems inside Q.

We next introduce a different, much simpler closed system Q${}^{\prime }$ consisting of three coupled harmonic oscillators. Again the Hilbert space $H^{\left(Q^{\prime }\right)}$ has a discrete countable basis, and if we further impose an upper energy limit, we can arrange for $\dim H^{\left(Q\right)}=\dim H^{\left(Q^{\prime }\right)}$. The two Hilbert spaces are therefore isomorphic, and there exists an isomorphism map for which the initial Q${}^{\prime }$ state corresponds to the initial Q state. This means we can effectively regard Q and Q${}^{\prime }$ as the *same* system with the same initial state $|\Psi_{0}\rangle$ evolving under different time evolutions U(t) and V(t). Variables $B_{k}$ for Q${}^{\prime }$ are different operators in $H^{\left(Q\right)}$, corresponding to the oscillator positions and momenta, etc. With respect to the alternate V(t) evolution, the expectations of these Q${}^{\prime }$ variables would be

(5) $${\langle B_{k}\rangle }_{t}^{\prime }=\langle \Psi_{0}|V^{†}\left(t\right)B_{k}V|\Psi_{0}\rangle .$$

These expectations would tell us “what happens” in Q${}^{\prime }$. (The actual evolution of ${\langle B_{k}\rangle }_{t}$ under the actual time evolution U(t) would, of course, be quite different.)

Now, consider a new set of variables in Q:

(6) $${\tilde{B}}_{k}=\left(U\left(t\right)V^{†}\left(t\right)\right)B_{k}\left(V\left(t\right)U^{†}\left(t\right)\right).$$

The ${\tilde{B}}_{k}$ operators are time dependent. However, consider how their expectations evolve in time under the actual time evolution of Q:

$$\begin{array}{ccc} {\langle {\tilde{B}}_{k}\rangle }_{t} & = & \langle \Psi_{0}|U^{†}{\tilde{B}}_{k}U|\Psi_{0}\rangle \\ & = & \langle \Psi_{0}|U^{†}UV^{†}B_{k}VU^{†}U|\Psi_{0}\rangle \\ & = & \langle \Psi_{0}|V^{†}B_{k}V|\Psi_{0}\rangle , \end{array}$$

exactly the time dependence of ${\langle B_{k}\rangle }_{t}^{\prime }$ under the alternate Q${}^{\prime }$ time evolution V. In other words, with respect to these time-dependent variables, the complex system Q behaves exactly like the much simpler system Q${}^{\prime }$.

There is nothing particularly strange about considering time-dependent observables. We have described Q and its evolution using the *Schrödinger picture* [11], in which observables are typically time-independent and system states evolve in time. However, we can also use the equivalent (and only slightly less familiar) *Heisenberg picture*, in which time dependence is shifted to the observables. (The time-dependence of observables in the Heisenberg picture has conceptual appeal. After all, to measure a particle’s spin on Monday or on Tuesday would require slightly different experimental set-ups, and so the two observables may plausibly be represented by different operators.) The system state is thus $|\Psi_{0}\rangle$ at all times, but the observables are redefined as

(7) $${\hat{A}}_{k}\left(t\right)=U^{†}\left(t\right)A_{k}U\left(t\right).$$

Then, ${\langle A_{k}\rangle }_{t}=\langle \Psi (t)|A_{k}|\Psi (t)\rangle =\langle \Psi_{0}|{\hat{A}}_{k}\left(t\right)|\Psi_{0}\rangle$. In perturbation theory, we also frequently use an *interaction picture*, in which the time evolution due to an unperturbed Hamiltonian $H_{0}$ is shifted to the observables, while the interaction Hamiltonian $H_{\mathrm{int}}$ produces changes in the system state.

**Figure 1:**
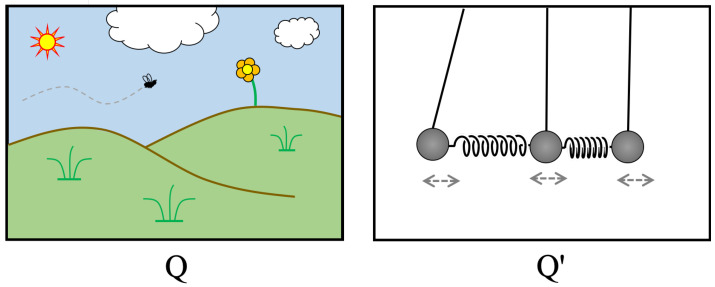
Two universes. Q is complex and contains many subsystems, including those that may be regarded as observers (such as the bee). Q${}^{\prime }$ is extremely simple. Nevertheless, the two Hilbert spaces $H^{\left(Q\right)}$ and $H^{\left(Q^{\prime }\right)}$ are isomorphic, so that Q and Q${}^{\prime }$ may be regarded as two pictures of the *same* universe.

What we have done, therefore, is simply changed pictures. With respect to the time-dependent variables ${\tilde{B}}_{k}\left(t\right)$ in the *Q${}^{\prime }$ picture*, the actual time evolution of Q exactly matches the hypothetical time evolution of Q${}^{\prime }$. In addition, of course, we can generalize this idea. For *any* closed Q${}^{\prime }$ with a Hilbert space of the same dimension as $H^{\left(Q\right)}$, and for any hypothetical Q${}^{\prime }$ time evolution V(t), we can find a set of time-dependent variables with respect to which the actual Q time-evolution looks like the alternate Q${}^{\prime }$ evolution. Complex universes can be made to look simple and vice versa. See Figure 1.

### 1.3. Grue and Bleen

Our argument calls to mind an idea from philosophy, devised in 1955 by Nelson Goodman [12]. We begin with familiar terms *blue* and *green* describing the colors of objects in our surroundings. Now, we fix a time *T* and define new terms *grue* and *bleen* as follows:

- An object is *grue* if is *green* before *T* and *blue* after.
- An object is *bleen* if it is *blue* before *T* and *green* after.

Goodman presented this idea to illustrate his “new riddle of induction”. If we fix *T* to lie in the future, then all present evidence that an object is *green* is also evidence that it is *grue*. Here, however, we are not principally concerned about inductive reasoning. It does not matter to us whether *T* lies in the future or the past.

In the quantum situation, the ordinary Q-observables $A_{k}$ correspond to the ordinary colors *green* and *blue*. The time-dependent Q${}^{\prime }$-picture observables ${\tilde{B}}_{k}$ correspond to the new terms *grue* and *bleen*.

We have an intuition that the terms *grue* and *bleen* are less basic than *green* and *blue*. After all, the definitions of *grue* and *bleen* are explicitly time-dependent. On the other hand, suppose we start with *grue* and *bleen* and pose these time-dependent definitions:

- An object is *green* if it is *grue* before *T* and *bleen* after.
- An object is *blue* if is it *bleen* before *T* and *grue* after.

Thinking only about the language, the best we can do is say that the *green-blue* system and the *grue-bleen* system are time-dependent *relative to each other*.

In the same way, we could begin with the ${\tilde{B}}_{k}$ description and define the Q-picture $A_{k}$ operators as time-dependent combinations of them. Each set of observables is time-dependent with respect to the other.

We can distinguish the two color systems by going outside mere language and considering the operational meaning of the terms. We can define green and blue by a measurement of, say, light wavelength. To determine whether an object is green, we can use a similar operational procedure both before and after time *T*. However, the procedure to determine whether the object is grue will work differently before and after *T*. It is this appeal to external facts that makes the green-blue distinction more basic and elementary than the grue-bleen distinction.

What can we say about our Q and Q${}^{\prime }$ pictures? We might appeal to the physical measurement procedures required to measure $A_{k}$ and ${\tilde{B}}_{k}$. The procedure for measuring $A_{k}$ is simple and time-independent, while that for measuring ${\tilde{B}}_{k}$ is complicated and changes with time. However, as long as we only consider measurement devices and processes within our closed quantum system, this does not suffice. ${\tilde{B}}_{k}$ devices and processes would be simple and time-independent in the Q${}^{\prime }$ picture, while $A_{k}$ devices and processes would be wildly time-varying in the same picture.

This is a reference frame problem. In both Galilean and Einsteinian relativity, there is no natural, universal way to identify points in space at different times. Space is too smooth and uniform; it does not have intrinsic “landmarks”. Hence, there is no natural and universal way to determine whether an object is “at rest”. In the same way, the Hilbert space $H^{\left(Q\right)}$ is also too smooth and uniform to identify state vectors and operators at different times. From within the system, we cannot determine whether a given collection of observables is time-dependent.

If we cannot distinguish the Q and Q’ pictures from within the system, the natural thing is to appeal to hypothetical measurement devices external to Q, unaffected by our change of picture. Then, $A_{k}$ devices are objectively simpler than ${\tilde{B}}_{k}$ devices. However, this appeals to something *outside* of the closed system Q is explicitly excluded by DeWitt’s maxim. We appear to be left with an inescapable dilemma. If we can only consider how the state of the system evolves, then that same history $|\Psi (t)\rangle$ can appear, with respect to different pictures, as either the complex system Q or the simple system Q${}^{\prime }$ *or any other quantum system with the same Hilbert space, undergoing any unitary time evolution whatsoever*. We cannot identify one of these pictures as the “correct” one without appealing to external measurement devices—that is, to measurement apparatus not treated as part of the isolated quantum system.

### 1.4. What Is a System?

Since the Hilbert spaces of quite different quantum systems are isomorphic, some additional information is required to apply quantum theory in an unambiguous way. This is not a novel point. For example, David Wallace [13] says, “[A]bsent additional structure, a Hilbert-space ray is just a featureless, unstructured object, whereas the quantum state of a complex system is very richly structured.” Wallace regards this additional structure as part of the specification of the quantum system in the first place. He considers two possible ways to provide this structure: a specified decomposition of the quantum system into subsystems (and thus its Hilbert space into quotient spaces), or a specified set of operators of fixed meaning. In this view, the two universes Q and Q’, with sets of operators $\left\{A_{k}\right\}$ and $\left\{{\tilde{B}}_{k}\right\}$, are entirely different systems rather than different pictures of the same system.

The rest of this paper has two aims: first, we want to pin down the nature of the additional structure that Wallace posits. We will do this by considering the problem in more generality. Section 2 presents a general framework for describing theories that include states, time evolution, and interpretational statements. Such a framework naturally entails groups of automorphisms, which we examine in Section 3. Some theories, including both quantum and classical mechanics, require “frame information” to resolve ambiguities that arise from these automorphisms. Section 4 presents several examples of our framework in action.

In Section 5, we turn to our second aim, which is to use our general framework to evaluate the additional structure required for a meaningful interpretation (of the many-worlds variety or not). What this physical nature of this frame information? In what ways might the strict many-worlds program—as embodied by De Witt’s maxim—prove inadequate? Section 6 includes remarks and observations occasioned by our line of reasoning.

## 2. A General Framework

### 2.1. States and Time Evolution

A *schema* for a theory has several parts. We begin with a set of **states** S={x,y,z,…}. Informally, these might be definite states or, in the case of a non-deterministic theory, probability distributions over collections of definite states.

To model time evolution, we introduce a sequence $\left(t_{0},t_{1},\ldots ,t_{N}\right)$ of times, where N≥1. Each time $t_{k}$ is associated with a state $x_{k}=x\left(t_{k}\right)\in S$. The whole sequence $\vec{x}=\left(x_{0},x_{1},\ldots ,x_{N}\right)$ may be termed a *trajectory*. Our schema includes a set of **kinematically possible maps** K={D,E,…}, which are functions on the set of states: D:S→S for D∈K. (To avoid a proliferation of parentheses, we will denote the action of *D* on state *x* as Dx rather than D(x).) The maps in K describe the evolution of the state over each interval in our time sequence. Thus, for the interval from $t_{k}$ to $t_{k+1}$,

(8) $$x_{k+1}=D_{k+1,k}\;x_{k}$$

for some $D_{k+1,k}\in K$. The sequence $\vec{D}=\left(D_{1,0},D_{2,1},\ldots ,D_{N,N-1}\right)$ thus describes the time evolution over the entire sequence of time intervals. A pair $\left(x_{0},\vec{D}\right)$ includes an initial state $x_{0}\in S$ and a sequence $\vec{D}\in K^{N}$ of time evolution maps; such a pair is called a specific *instance* of the theory.

We can of course compose successive maps. In the general case, we do not assume that K is closed under composition, so it may be that $D_{k+2,k}=D_{k+2,k+1}D_{k+1,k}$ is not in K. However, in many specific cases, K actually forms a group, being closed under composition and containing both the identity map 1 and inverses for every element. In such cases, we say that our theory is *reversible*. In a reversible theory, K includes maps between any pair of times $t_{j}$ and $t_{k}$, where j,k∈{0,…,N}:

(9) $$D_{k,j}=\left\{\begin{array}{cc} D_{k,k-1}\cdots D_{j+1,j} & k>j\\ 1 & k=j\\ D_{j,k}^{-1} & k<j. \end{array}\right.$$

The algebraic structure of K is reflected in the way that maps combine. If K is a group, then for any j,k,l∈{0,…,N}, we have

(10) $$D_{k,j}=D_{k,l}D_{l,j}.$$

(Note that, in a reversible theory, this relation holds for any time order of $t_{j}$, $t_{k}$ and $t_{l}$.)

If K is a group, it is not hard to generalize our schema to a continuous time variable *t*. A trajectory is a function x(t) that yields a state in S at any time. For any two times $t_{1}$ and $t_{2}$, we have a map $D(t_{2},t_{1})$ such that $x\left(t_{2}\right)=D\left(t_{2},t_{1}\right)x\left(t_{1}\right)$. These maps are related to one another by a composition relation analogous to Equation (10):

(11) $$D\left(t_{2},t_{1}\right)=D\left(t_{2},t_{3}\right)D\left(t_{3},t_{1}\right).$$

Everything in the schema works pretty much the same. For ease of exposition, we will base our discussion on a finite sequence of discrete times $\left(t_{0},\ldots ,t_{N}\right)$, leaving the straightforward generalization to continuous time schemata for the reader.

At the other end of the “time complexity spectrum”, our later examples of our framework will involve only a single time interval from $t_{0}$ to $t_{1}$. The set K may still be closed in these schemata, or even have a group structure, but the composition of maps will not correspond to time evolution over successive intervals.

### 2.2. Interpretational Statements

What is an interpretation? To give a general answer to this question is beyond the scope of this paper. We will merely assume that every theory comes equipped with a collection I of **interpretational statements**, which are propositions about the state and/or the map of a particular instance of the theory. For example, immediately after giving the quantum state in Equation (2), we stated, *The memory record of the observer (“up” or “down”) has become correlated to both the original spin and the reading on the apparatus.* This is an interpretational statement, and its truth is determined by the properties of the state in Equation (2). In our abstract framework, we will not be much concerned with the *content* of an interpretational statement, but rather with the fact that it is a statement about elements of the mathematical formalism of our theory. Thus, a state proposition is a statement P(x) about a state x∈S, and a more general type of proposition would be $P(x_{0},\vec{D})$, referring to an initial $x_{0}\in S$ and a sequence of time evolution maps $\vec{D}$. (Notice that the more general form also encompasses propositions about states at any time $t_{k}$, since we can construct the entire state trajectory $\vec{x}$ from $x_{0}$ and $\vec{D}$.) Statements of both kinds may appear in I. Whatever else an interpretation may include, it must surely entail such a set of interpretational statements; and if this set is empty or trivial, the interpretation is nugatory.

An interpretational statement is either true or not true. We say “not true” here rather than “false” because it may be that a statement has an indeterminate value. Consider a naive example. For a spin-1/2 particle, our statement *P* is “$S_{z}=+\frac{\hbar }{2}$.” If the spin state is $|↑\rangle$, the statement *P* is true, inasmuch as a measurement will surely confirm it. If the spin state is $|↓\rangle$, it is reasonable to call *P* false, since its negation (“$S_{z}\neq +\frac{\hbar }{2}$”) is true in the same sense. However, if the spin state is $|\to \rangle$, neither *P* nor its negation is true. Thus, we simply say that *P* is true for the state $|↑\rangle$ and not true for other states like $|↓\rangle$ and $|\to \rangle$.

Without a more explicit “theory of interpretation”, we cannot say more about the structure of I. For example, we do not assume that the collection I has any particular algebraic closure properties. If P,Q∈I, we have no warrant to declare that ¬P, P∨Q, or P∧Q are part of I.

## 3. Similarities

### 3.1. Simple Similarities

There is one more essential element to our schema. It may be that some states in S are equivalent to others. That is, some states will yield exactly the same true (or not true) interpretational statements. Thus, we suppose that our schema comes equipped with a set U of *K-similarities* (or just *similarities*). Each similarity is a map V:S→S that satisfies the following property:

**Property S.** Both of these are true of *V*:

- *V* is a bijection.
- $VDV^{-1}\in K$ if and only if D∈K.

We do *not* assume that every *V* with this property is necessarily a similarity in U. However, we note that, if *V* and *W* satisfy Property S, so does VW and $V^{-1}$. Thus, it is natural to suppose that the collection U forms a group, and we will make that assumption.

Think of the K-similarity map V∈U as a set of “spectacles” with which we examine the states in S. Through the spectacles, the state *x* appears to be the state $\tilde{x}=Vx$. The dynamical law that applies the kinematically possible map *D* to *x* appears to be a different map $\tilde{D}=VDV^{-1}$, which is also in K:

(12) x0⟶Vx˜0D1,0↓D1,0D˜1,0↓D˜1,0x1⟶Vx˜1D2,1↓D2,1D˜2,1↓D˜2,1⋮⋮DN,N−1↓DN,N−1D˜N,N−1↓D˜N,N−1xN⟶Vx˜N

The point is that $\left({\tilde{x}}_{0},\tilde{\vec{D}}\right)$ is an instance of our theory if and only if $\left(x_{0},\vec{D}\right)$ is. The situation viewed through the spectacles fits the schema just as well as the situation without. The spectacles simply provide a new “frame of reference” for describing the state and the time evolution.

If the theory is reversible, so that every E∈K has an inverse map $E^{-1}$, we note that every element E∈K automatically satisfies Property S: *E* is a bijection, and $EDE^{-1}\in K$ if and only if D∈K. This opens up the possibility that the K-similarity group U might contain (among other things) every map in K. If K⊆U, we say that the the K-similarity group U is *K-inclusive*.

A K-similarity is not at all the same thing as a dynamical symmetry of a particular instance of the theory. If *D* is a particular dynamical map, a dynamical symmetry *V* would satisfy VD=DV, which in turn implies that $VDV^{-1}=D$. Property S instead has a weaker condition that $\tilde{D}=VDV^{-1}$ is some map in K; but this condition must hold for every map D∈K. From a slightly different point of view, the similarity map *V* acts a symmetry of the *sets* S and K, in that VS=S and $VKV^{-1}=K$.

Interpretational statements must respect similarities within the schema. For instance, suppose P(x) is a state proposition in I. Then, for any V∈U, we must have P(x)⇔P(Vx) (by which we mean that P(x) and P(Vx) are true for exactly the same states x∈S). For a more general type of proposition,

(13) $$P\left(x_{0},\vec{D}\right)\Leftrightarrow P\left({\tilde{x}}_{0},\tilde{\vec{D}}\right)=P\left(Vx_{0},\left(VD_{1,0}V^{-1},\ldots ,VD_{N,N-1}V^{-1}\right)\;\right)$$

for all V∈U. Each similarity V∈U imposes a restriction on the possible interpretational statements in I. Therefore, we can regard I and U as “dual” to one another. The larger the set of K-similarities, the more restricted is the allowed set of interpretational statements.

### 3.2. Extended Similarities

The similarities V∈U are spectacles with which we may view an instance of our theory. However, it is also possible to imagine time-dependent spectacles which apply different maps at different times. This is analogous to translating from *blue-green* color language to *grue-bleen* language.

What kind of time-dependent spectacles might we have? An *extended similarity map* is a sequence $\vec{V}=\left(V_{0},V_{1},\ldots ,V_{N}\right)$ of maps on S. We require that this sequence satisfies the following property:

**Property S${}^{\left(\mathrm{ext}\right)}$.** Both of these are true of all maps in $\vec{V}$:

- $V_{k}\in U$.
- $V_{k+1}DV_{k}^{-1}\in K$ if and only if D∈K.

The meaning of this property can be explained by a diagram.

(14) x0⟶V0x˜0D1,0↓D1,0D˜1,0↓D˜1,0x1⟶V1x˜1D2,1↓D2,1D˜2,1↓D˜2,1⋮⋮DN,N−1↓DN,N−1D˜N,N−1↓D˜N,N−1xN⟶VNx˜N

Property S${}^{\left(\mathrm{ext}\right)}$ therefore requires that, for an extended similarity $\vec{V}$, $\left({\tilde{x}}_{0},\tilde{\vec{D}}\right)$ is an instance of the theory if and only if $\left(x_{0},\vec{D}\right)$ is. We may regard $\vec{V}$ as a symmetry of the sets S and $K^{N}$, in the sense that $V_{k}S=S$ and $V_{k+1}KV_{k}^{-1}=K$ for all *k*.

We denote the set of extended similarities by $U^{\left(\mathrm{ext}\right)}$. We do not assume that every extended map $\vec{V}$ satisfying Property S${}^{\left(\mathrm{ext}\right)}$ must be in $U^{\left(\mathrm{ext}\right)}$. It is interesting to note that, in some schemata, there are examples in which $V_{k}\in U$ for all *k*, but $\vec{V}$ fails to satisfy Property S${}^{\left(\mathrm{ext}\right)}$. However, if *V* satisfies Property S, then (V,V,…,V) must also satisfy Property S${}^{\left(\mathrm{ext}\right)}$. Therefore, we will assume $\left(V,V,\ldots ,V\right)\in U^{\left(\mathrm{ext}\right)}$ for every V∈U. That is, time-independent spectacles are always allowed in $U^{\left(\mathrm{ext}\right)}$, and in this sense we may say that $U\subseteq U^{\left(\mathrm{ext}\right)}$. We further assume that the set $U^{\left(\mathrm{ext}\right)}$ of extended similarities is itself a group.

An element $\vec{V}$ in the extended similarity group $U^{\left(\mathrm{ext}\right)}$ turns one instance $\left(x_{0},\vec{D}\right)$ of a theory into another instance $\left({\tilde{x}}_{0},\tilde{\vec{D}}\right)$ of the theory. However, in a more fundamental sense, we should regard $\left(x_{0},\vec{D}\right)$ and $\left({\tilde{x}}_{0},\tilde{\vec{D}}\right)$ merely as different *pictures* of the same actual situation, the one picture transformed into the other by the use of (possibly time-dependent) spectacles. Of course, the truth of an interpretational statement should not depend on the picture used to describe the instance of the theory. Thus, we require that

(15) $$P\left(x_{0},\vec{D}\right)\Leftrightarrow P\left({\tilde{x}}_{0},\tilde{\vec{D}}\right)=P\left(V_{0}x_{0},\left(V_{1}D_{1,0}V_{0}^{-1},\ldots ,V_{N}D_{N,N-1}V_{N-1}^{-1}\right)\;\right)$$

for each P∈I and $\vec{V}\in U^{\left(\mathrm{ext}\right)}$. We recognize this as just the extended version of Equation (13), and we note that it includes that fact as a special case.

We note that any extended similarity $\vec{V}$ preserves the composition relations among the maps in $K^{N}$. Suppose for simplicity that our theory is reversible, and we specify a particular sequence of evolution maps $\vec{D}=\left(D_{1,0},D_{2,1},\ldots ,D_{N,N-1}\right)$. We define the maps $D_{kj}$ according to Equation (9) and say that ${\tilde{D}}_{kj}=V_{k}D_{kj}V_{j}^{-1}$. Then, the transformed set of maps satisfies a transformed version of Equation (10), namely that

(16) $${\tilde{D}}_{k,j}={\tilde{D}}_{k,l}{\tilde{D}}_{l,j}$$

for any j,k,l∈{0,…,N}. In other words, $\vec{V}$ preserves the algebraic structure of K that arises from time evolution over successive time intervals.

### 3.3. The DeWitt Principle

Our framework tells us that an interpretational system involves, not simply the set I of interpretational statements, but also the group $U^{\left(\mathrm{ext}\right)}$. The former includes everything that might be truthfully asserted about a physical situation. The latter tells us which different instances $\left(x_{0},\vec{D}\right)$ and $\left({\tilde{x}}_{0},\tilde{\vec{D}}\right)$ of a theory should be regarded as different pictures of the same situation. These are related, since the same interpretational statements must be true in both equivalent pictures.

DeWitt’s maxim says that the interpretation of quantum theory can be derived from the mathematical structure of the theory. For this to hold, we must be able to derive I and $U^{\left(\mathrm{ext}\right)}$ from the mathematical structure of S and K. No outside elements or special assumptions need be, or should be, introduced.

Therefore, *every* map *V* that satisfies Property S is a symmetry of S and K, and so should be included in U; and the same is true of every sequence $\vec{V}$ of such maps satisfying Property S${}^{\left(\mathrm{ext}\right)}$. Thus, we pose the following **principle of maximal similarity**, which we may, for convenience, call the “DeWitt Principle”.

**DeWitt Principle.** For a given S and K, we must choose the similarity group U and the extended group $U^{\left(\mathrm{ext}\right)}$ to be maximal.

That is,

- The similarity group U contains every map *V* satisfying Property S.
- The extended similarity group $U^{\left(\mathrm{ext}\right)}$ contains every sequence $\vec{V}$ of elements of U satisfying Property S${}^{\left(\mathrm{ext}\right)}$.

It is not hard to show that the maximal U and $U^{\left(\mathrm{ext}\right)}$, as defined, exist and are groups.

When we assume that U and $U^{\left(\mathrm{ext}\right)}$ are maximal, we maximally constrain the set I of interpretational statements. This is the other side of the DeWitt Principle. If the mathematical formalism of a theory is capable of yielding its own interpretation, it follows that the only allowable interpretational statements are those that can be derived from the mathematical formalism alone. These interpretational statements must “look the same” through both time-independent and time-dependent similarity spectacles.

Of course, as we will see, it may be that the appropriate choice of $U^{\left(\mathrm{ext}\right)}$ is not maximal. There may be additional constraints on similarities, allowing for a wider range of interpretational statements. However, a non-maximal choice of $U^{\left(\mathrm{ext}\right)}$ cannot be derived from the structure of the sets S and K.

### 3.4. Reversibility, Transitivity, and Interpretation

Suppose we have a reversible theory, so that K is a group. Then, the DeWitt Principle implies that every element of K is also a K-similarity in U. Thus, U is K-inclusive (i.e., K⊆U). In addition, in fact, we can say more. In a reversible theory, for any sequence $\vec{E}=\left(E_{0},E_{1},\ldots ,E_{N}\right)\in K^{N}$ must be in $U^{\left(\mathrm{ext}\right)}$. Thus, $K^{N}\subseteq U^{\left(\mathrm{ext}\right)}$.

We say that the set K of kinematically possible maps acts *transitively* on the state set S if, for any x,y∈S, there exists D∈K so that y=Dx. That is, any given state *x* can be turned into any other given state *y* by some kinematically possible dynamical evolution.

Consider a reversible theory schema in which K acts transitively on S. As we have seen, the DeWitt Principle implies that K⊆U. Any such K-inclusive similarity group U must also act transitively on S. However, this has an important and baleful implication for the collection I of interpretational statements. Suppose *P* is a state proposition, and consider two arbitrary states x,y∈S. By transitivity, there exists V∈U such that y=Vx. Thus, P(x)⇔P(Vx)=P(y). In other words, the only possible state propositions in I are those that are true for every state or for none. There are no non-trivial state propositions in I.

The implications for the extended similarity group $U^{\left(\mathrm{ext}\right)}$ are even stronger. The DeWitt Principle applied to $U^{\left(\mathrm{ext}\right)}$ implies that $K^{N}\subseteq U^{\left(\mathrm{ext}\right)}$. This means we can freely choose $\vec{V}\in K^{N}$ and guarantee that $\vec{V}\in U^{\left(\mathrm{ext}\right)}$.

Now, choose any two states $x_{0},y_{0}\in S$ and any two sequences $\vec{D},\vec{E}\in K^{N}$. Since K acts transitively on S, we can find $V_{0}\in K$ such that $y_{0}=V_{0}x_{0}$. Furthermore, for k≥1, the map $V_{k}=E_{k,k-1}V_{k-1}D_{k-1,k}\in K$, and so the sequence $\vec{V}$ forms “time-dependent spectacles” in $U^{\left(\mathrm{ext}\right)}$. The following diagram commutes:

(17) x0⟶V0y0D1,0↓D1,0E1,0↓E1,0x1⟶V1y1D2,1↓D2,1E2,1↓E2,1⋮⋮DN,N−1↓DN,N−1EN,N−1↓EN,N−1xN⟶VNyN

*Any specific instance $\left(x_{0},\vec{D}\right)$ of our theory can be transformed into any other specific instance $\left(y_{0},\vec{E}\right)$.* Therefore, the general interpretational statements $P(x_{0},\vec{D})$ and $P(y_{0},\vec{E})$ must both be equivalent. This may be stated as our main general result:

**Theorem** **1.** *Consider a reversible theory schema in which K acts transitively on S. If the DeWitt Principle holds, then I contains no non-trivial statements.*

We might restate this conclusion in another way: A reversible theory in which any state could in principle evolve to any other state *cannot* yield its own non-trivial interpretation without additional constraints on $U^{\left(\mathrm{ext}\right)}$.

## 4. Examples

In this section, we will set up a few examples of theory schemata and discuss some of the properties of each. For simplicity, each example considers time evolution over a single interval of time from $t_{0}$ to $t_{1}$.

### 4.1. Deck Shuffling

Consider a standard deck of 52 cards. The state set S consists of every arrangement of the cards in the deck, and a kinematically possible map is simply a permutation of the deck. All such permutations are in K.

Suppose now we divide the deck into two half-decks of 26 cards each. Every rearrangement of the whole deck is in S. However, our kinematically possible maps include only separate rearrangements of the half-decks. Thus, if the queen of hearts starts out in half-deck #1, it will stay there no matter what “time evolution” D∈K occurs. This, like the full-deck theory, is a reversible theory.

The DeWitt Principle implies that K⊆U for both theories. For the undivided deck, the permutation group acts transitively on the state set. This theory, therefore, has no non-trivial statements in I.

What about U and $U^{\left(\mathrm{ext}\right)}$ for the half-deck theory? In this schema, there are maps in the maximal U that are not in K. For instance, consider a map *X* on states that exchanges the two half-decks. This is not in K, but it does satisfy Property S since both $XDX^{-1}$ and $X^{-1}DX$ are half-deck shuffles. (The two half-decks are exchanged twice.) From the DeWitt Principle, both *X* and the identity map 1 are in U. However, the sequence $\vec{V}=\left(1,X\right)$ does not satisfy Property S${}^{\left(\mathrm{ext}\right)}$ and therefore is not in $U^{\left(\mathrm{ext}\right)}$.

In the half-deck theory, K is a group, but it does not act transitively on S. The divided deck with separate half-deck permutations does potentially have non-trivial statements in I. For example, the statement “All of the jacks are in the same half-deck” will not change its truth value if the half-decks are reshuffled or exchanged. Such a statement expresses a property that may be the basis for an interpretational statement.

### 4.2. Symbolic Dynamics

A very interesting example arises from *symbolic dynamics*. In symbolic dynamics, the states are bi-infinite sequences of symbols from a finite alphabet. The set of allowed sequences may be constrained by some rule; for instance, we may be restricted to binary sequences that never include more than two 1’s in succession. The particular example we will consider includes all binary sequences in S. This is known in the literature as the “full shift” and is the symbolic dynamics associated with the “baker’s map” on the unit square.

The dynamical maps are finite left or right shifts of the sequences in S. There are thus two reasonable choices for K. First, K might contain only the elementary map σ that shifts the sequence by one place: given a sequence $\vec{x}$, ${\left(\sigma \vec{x}\right)}_{i}=x_{i+1}$. Second, we might posit that K includes all finite shifts, so that $K=\{\ldots ,\sigma^{-1},1,\sigma ,\sigma^{2},\ldots \}$. This amounts to assuming that the underlying time evolution can occur at any finite speed, so that an arbitrary number of elementary shifts in either direction may occur within our given time interval.

We will make the second choice, which makes K a group and the theory reversible. Thus, under the DeWitt Principle, all the shifts in K are also similarities in the maximal group U. This maximal U also includes many other maps as well. For example, U contains the map β that complements the sequence: ${\left(\beta \vec{x}\right)}_{i}={\bar{x}}_{i}$, where $\bar{0}=1$ and $\bar{1}=0$. It also contains the reflection map ρ: ${\left(\rho \vec{x}\right)}_{i}=x_{-i}$. However, U cannot contain any map *V* that takes a constant sequence to a non-constant sequence.

Let us prove this assertion. Our definition of the similarity group U for symbolic dynamics implies the following:

If V∈U, then, for all n∈Z, there exists m∈Z such that $V^{-1}\sigma^{n}V=\sigma^{m}$, or equivalently $\sigma^{n}V=V\sigma^{m}$.

We will use the contrapositive of this fact.

If there exists n∈Z such that, for all m∈Z, we have $\sigma^{n}V\neq V\sigma^{m}$, then V∉U.

Now consider the constant sequence $\vec{b}=\ldots bbbb\ldots$, and suppose $V\vec{b}$ is not constant. Then, there exists n∈Z such that $\sigma^{n}V\vec{b}\neq V\vec{b}$. However, for any m∈Z, $\vec{b}=\sigma^{m}\vec{b}$, and so $\sigma^{n}V\vec{b}\neq V\sigma^{m}\vec{b}$. Thus, $\sigma^{n}V\neq V\sigma^{m}$, and hence V∉U.

The similarity group U does not act transitively on S. Therefore, even if we impose the DeWitt Principle, the statements in I may still include nontrivial statements like, “The sequence is constant”, which retain their truth value under shifts, reflection, complementation, etc.

### 4.3. Classical Hamiltonian Dynamics

Suppose we have a classical system described by a phase space with *n* real coordinates $q_{k}$ and *n* associated momenta $p_{k}$. To make things a bit simpler, we can shift our time coordinate so that $t_{0}=0$ and $t_{1}=\tau$. The allowed time evolutions in K are the “Hamiltonian maps” that result from a (possibly time-dependent) Hamiltonian function $H(q_{k},p_{k},t)$ acting over the time interval (t=0 to t=τ), so that

(18) $${\dot{p}}_{k}=\frac{dp_{k}}{dt}=-\frac{\partial H}{\partial q_{k}}\;\mathrm{and}\;{\dot{q}}_{k}=\frac{dq_{k}}{dt}=\frac{\partial H}{\partial p_{k}}.$$

Two maps can be composed as follows. Suppose we have maps $D_{1}$ and $D_{2}$, which are produced by Hamiltonian functions $H_{1}\left(q_{k},p_{k},t\right)$ and $H_{2}\left(q_{k},p_{k},t\right)$ controlling the dynamics over the time interval 0 to τ. Then, we can construct a new map $D_{21}$ via the following Hamiltonian:

(19) $$H_{21}\left(q_{k},p_{k},t\right)=\left\{\begin{array}{cc} 2H_{1}\left(q_{k},p_{k},2t\right) & 0\leq t\leq \tau /2\\ 2H_{2}\left(q_{k},p_{k},2t-\tau \right) & \tau /2<t\leq \tau . \end{array}\right.$$

This will cause the system to evolve according to a “two times faster” version of $H_{1}$ for the first half of the time interval, and a “two times faster” version of $H_{2}$ for the second half of the interval. The resulting change in state will simply be the map $D_{21}=D_{2}D_{1}$.

This theory is reversible, since the evolution by $H(q_{k},p_{k},t)$ can be exactly reversed by the Hamiltonian $-H(q_{k},p_{k},\tau -t)$. Thus, the maximal U includes all of K, and potentially many other maps.

The set of Hamiltonian maps also acts transitively on the classical phase space. Given any two points $\left(q_{k},p_{k}\right)$ and $\left(q_{k}^{\prime },p_{k}^{\prime }\right)$, it is not hard to write down a Hamiltonian function that evolves one into the other in the time interval from 0 to τ. Thus, if the DeWitt Principle holds, I contains no non-trivial interpretational statements.

### 4.4. Unitary Quantum Mechanics

In quantum theory, the states in S are vectors $|\psi \rangle$ of unit norm in a Hilbert space H. As before, we take dimH to be finite, though maybe extremely large. The kinematically possible maps K include all unitary operators on H. All such operators can be realized by evolving the state vector via the Schrödinger equation using the Hamiltonian operator H(t):

(20) $$i\hbar |\psi (t)\rangle =H\left(t\right)|\psi (t)\rangle \;⟹\;|\psi (t_{1})\rangle =U|\psi (t_{0})\rangle .$$

Since this theory is reversible, the maximal similarity group U includes all of the unitary operators in K. The unitary operators also act transitively on the unit vectors in a Hilbert space H. Thus, the DeWitt Principle excludes all non-trivial interpretational statements from I.

From these examples, we may draw a general lesson. Some theories have non-trivial statements whose truth value is unchanged by any similarity, even when U and $U^{\left(\mathrm{ext}\right)}$ are maximal. In this way, it is possible that “the mathematical formalism" of a theory could yield “its own interpretation”. However, this is impossible for many interesting theories, including both classical Hamiltonian dynamics and unitary quantum mechanics.

## 5. Taming Quantum Similarities?

Suppose we have a reversible theory schema in which K acts transitively on S. Under the DeWitt Principle, the unlimited similarity groups U and $U^{\left(\mathrm{ext}\right)}$ are too big to admit non-trivial interpretational statements in I. Therefore, any meaningful interpretation for the theory will require us to limit the similarity groups in some way. We must either have K¬⊆U or $K^{N}\neg \subseteq U^{\left(\mathrm{ext}\right)}$, or both. This is precisely the “additional structure” posited by Wallace [13], discussed in Section 1.4 above.

The basis for a limitation of this kind cannot be found in the mathematical formalism of S and K. Any such external limitation will therefore contravene our version of the DeWitt Principle. It will be useful here briefly to describe a couple of plausible “non-DeWitt” limitations on U and $U^{\left(\mathrm{ext}\right)}$ for the example of unitary quantum mechanics over a single time interval.

### 5.1. Subsystem Decomposition

First, suppose H can be decomposed as a tensor product of smaller spaces: $H=H^{\left(1\right)}⊗H^{\left(2\right)}⊗\cdots ⊗H^{\left(n\right)}$. (This is one of the possibilities mentioned by Wallace.) Each $H^{\left(k\right)}$ represents the state space of a subsystem of the whole quantum system. This does not by itself limit the kinematically possible time evolutions in K, since the subsystems might interact with one another in an arbitrary way. However, if we take the subsystem decomposition as given, we may plausibly restrict our similarities to operators of the form:

(21) $$V=V^{\left(1\right)}⊗V^{\left(2\right)}⊗\cdots ⊗V^{\left(n\right)}.$$

Our similarity spectacles can modify the states of the individual subsystems, but they cannot mix the subsystems together. In this case, even though K acts transitively on S, the similarity group U does not. This restriction on U (and hence $U^{\left(\mathrm{ext}\right)}$) allows for many non-trivial interpretational statements in I. For example, consider the state proposition P(x) = “In state *x*, subsystems 1 and 2 are entangled.” Since the K-similarities do not mix subsystems, this statement has the same truth value, regardless of what similarity spectacles are applied to the state.

We must remember, however, that there are infinitely many tensor product decompositions of H [14]. That is, we can decompose a composite system into subsystems in an unlimited number of ways. States that are entangled with respect to one decomposition may not be entangled with respect to another. For instance, consider a system with dimH=4 that can be regarded as a pair of qubits, labeled 1 and 2. This pair could be in one of the four entangled “Bell states”:

(22) $$\begin{array}{c} |\Phi_{\pm }^{\left(12\right)}\rangle =\frac{1}{\sqrt{2}}\left(|0^{\left(1\right)}\rangle ⊗|0^{\left(2\right)}\rangle \pm |1^{\left(1\right)}\rangle ⊗|1^{\left(2\right)}\rangle \right)\\ |\Psi_{\pm }^{\left(12\right)}\rangle =\frac{1}{\sqrt{2}}\left(|0^{\left(1\right)}\rangle ⊗|1^{\left(2\right)}\rangle \pm |1^{\left(1\right)}\rangle ⊗|0^{\left(2\right)}\rangle \right). \end{array}$$

On the other hand, there exists an entirely different decomposition of the system into qubits designated A and B, with respect to which these are product states:

(23) $$\begin{array}{ccc} |\Phi_{+}^{\left(12\right)}\rangle =|\Phi^{\left(A\right)}\rangle ⊗|{+}^{\left(B\right)}\rangle \; & \; & |\Psi_{+}^{\left(12\right)}\rangle =|\Psi^{\left(A\right)}\rangle ⊗|{+}^{\left(B\right)}\rangle \\ |\Phi_{-}^{\left(12\right)}\rangle =|\Phi^{\left(A\right)}\rangle ⊗|{-}^{\left(B\right)}\rangle \; & \; & |\Psi_{-}^{\left(12\right)}\rangle =|\Psi^{\left(A\right)}\rangle ⊗|{-}^{\left(B\right)}\rangle . \end{array}$$

Subsystem decompositions are necessary to describe many important processes. For example, decoherence processes depend on the decomposition of the whole system into a subsystem of interest and an external environment.

We must therefore ask, where does a special subsystem decomposition come from? Neither the set of possible states S nor the set K of kinematically possible maps picks out a particular decomposition. It must come from somewhere else. Non-trivial interpretational statements about entanglement are only possible once a preferred decomposition is specified, by whatever means.

From the point of view espoused by Wallace [13], the subsystem decomposition is simply a *given* for a particular physical situation. The mathematical formalism of quantum theory specifies S and K*and* a similarity group U that respects the preferred subsystem decomposition. The question of the physical basis for this decomposition—its origin and representation in the state and dynamics of the system of interest—simply cannot arise. As Wallace himself points out, however, this decomposition is itself the real source of the complexity of the quantum world.

If we allow ourselves to invoke a hypothetical outside observer, it is easy to see how a preferred decomposition could emerge. The subsystems in the special decomposition correspond to different ways that the observer can access the system of interest. *This* sort of control or measurement interaction affects *this* subsystem, *that* sort affects *that* subsystem. The decomposition emerges from the nature of the devices that implement these operations. However, these devices do *not* reside in the system of interest, and their intervention means that the system is no longer isolated.

Subsystem decomposition is a special type of quantum reference frame information, called *meronomic* information [14]. We will briefly discuss the role of quantum reference frames in Section 5.3 below.

### 5.2. Time-Independent Spectacles

Here is another potential limitation, this one on the extended similarity group $U^{\left(\mathrm{ext}\right)}$. We allow any unitary map V∈U, but we declare that the only elements of $U^{\left(\mathrm{ext}\right)}$ are those of the form (V,V). Only “time-independent spectacles” are allowed; no “grue-bleen” pictures are permitted. In this case, U acts transitively on S, and only trivial state propositions P(x) are possible in I. However, there are non-trivial general propositions in I. For example, consider the statement Q(x,D)= “State *x* is a fixed point of dynamics *D*; that is, Dx=x.” If we apply the (time-independent) similarity map *V* to turn instance (x,D) into $\left(\tilde{x},\tilde{D}\right)$, we find that $\tilde{D}\tilde{x}=VDV^{-1}Vx=VDx=Vx=\tilde{x}$. The statement Q(x,D) might be true or not—it is not trivial—but in any case $Q\left(x,D\right)\Leftrightarrow Q\left(\tilde{x},\tilde{D}\right)$.

Even for a schema with a single time interval, we are effectively dealing with *two* sets of states: $S_{0}$ at $t_{0}$ and $S_{1}$ at $t_{1}$. These are of course both isomorphic to S. One connection between the sets is the dynamical evolution D∈K, which indicates which $x_{0}\in S_{0}$ evolves to $x_{1}\in S_{1}$. To claim that our spectacles are “time-independent” means that we have another canonical isomorphism between the two, which lets us identify which states in $S_{0}$ are taken to be *identical* to other states in $S_{1}$. We might denote this canonical isomorphism by the symbol 1, but this hides the fact that there are *infinitely many* possible isomorphisms between the two sets. To say unambiguously that a state at $t_{0}$ is the same state as another at $t_{1}$, or to define some spectacles as “time-independent”, we must invoke this second way (besides the time evolution map D∈K) to link together $S_{0}$ and $S_{1}$.

We might, of course, simply argue that this link between $S_{0}$ and $S_{1}$ is part of the *definition* of the system of interest. However, if we do not regard this answer-by-definition as satisfactory, the question remains: What is the physical origin of such a link, which is required to make the needed restrictions on $U^{\left(\mathrm{ext}\right)}$? If the quantum system is truly isolated, no satisfactory answer is possible, since *D* itself describes how all parts of the state evolve, and thus expresses everything about the dynamical connection between times $t_{0}$ and $t_{1}$. However, once again, a hypothetical outside observer can provide a plausible answer. The external apparatus of the observer can allow us to define what it means for a state to remain the same over time. In effect, it provides a fixed reference frame for the Hilbert space of states.

Such an explanation seems natural, but, of course, it invokes an observer that is *not* treated as part of the isolated quantum-mechanical system. It runs counter to the letter and spirit of DeWitt’s maxim.

### 5.3. Quantum Reference Frames

Ours is essentially a reference frame problem, so it is natural to ask whether the existing theory of quantum reference frames [15] can help resolve it. Unfortunately, it cannot.

In quantum reference frames, we begin with an abstract symmetry group G. Any system is made of up of elementary subsystems, each of which has its own unitary representation of G. The symmetry element g∈G is represented by the unitary operator

(24) $$V_{g}=V_{g}^{\left(1\right)}⊗V_{g}^{\left(2\right)}⊗\cdots ⊗V_{g}^{\left(N\right)}$$

for subsystems 1, …, N. These operators are dynamical symmetries for the system, so that the only available operations are symmetric ones, those that commute with $V_{g}$. Nevertheless, if part of the system is in an asymmetric state, we can use that state as a resource to perform asymmetric operations on other parts of the system. This asymmetric resource state constitutes a quantum reference frame.

To take an example, suppose our subystems are spin-1/2 particles and our symmetry group G is the set of rotations in 3D space. Each spin has its own SU(2) representation of this group. We can only perform rotationally invariant operations on the spins. A measurement of $S_{z}^{\left(1\right)}$ on spin #1 thus seems out of the question, since we cannot a priori specify the *z*-axis. However, suppose the remaining N-1 spins are provided in the state $|{↑}^{\left(k\right)}\rangle$, aligned with the (unknown) *z*-axis. Then, we can use these extra spins to perform a global rotationally invariant operation that approximates an $S_{z}^{\left(1\right)}$ measurement on the first spin. We have used the asymmetric $|{↑}^{\left(k\right)}\rangle$ states as a quantum reference frame resource.

The decomposition of a quantum system into subsystems can also be described as a quantum reference frame problem [14]. For example, suppose we consider some quantum systems with dimH=4 (called “tictacs” in [14]), and we wish to specify a particular subsystem decomposition for these into qubit pairs. We can do this by supplying additional tictacs in a special “asymmetric” state that encodes the subsystem division. For example, suppose we are considering a series of tictacs in state $|\Phi \rangle$, and we wish to estimate the Schmidt parameter of the entangled state for a particular qubit decomposition. We can accomplish this with the assistance of a supply of tictac pairs in the resource state $|\Psi_{-}^{\left(13\right)}\rangle ⊗|\Psi_{-}^{\left(24\right)}\rangle$ (where the first tictac is made up of qubits #1 and #2 and the second is made up of #3 and #4). If we specify how to decompose a particular system into subsystems, we say that we have provided *meronomic* frame information. We therefore see that meronomic information for dividing tictacs into qubits can be regarded as a kind of quantum information, information that can in principle be represented by the state of quantum systems.

The symmetry group G (or more precisely its unitary representation $\left\{V_{g}\right\}$) is somewhat analogous to our similarity group U. While the symmetry element *g* remains unknown, we can only make G-invariant statements about our system. Notice that, if we add new subsystems to our system, we do not actually enlarge the symmetry group. The symmetry group for N spins is still just a representation of SU(2). Informally, we may say that the “symmetry frame problem” stays essentially the same when we enlarge the system, but the additional pieces may provide asymmetric states as resources to help resolve the problem.

However, under the DeWitt Principle, the similarity group U for N spins contains all of $U(2^{N})$, the full set of unitary operators on the Hilbert space for the spins. The “similarity frame problem” gets *worse* as we add spins, not better. Even if we are somehow granted the subsystem decomposition between the spins, so that the similarity group contains U(2)⊗U(2)⊗⋯⊗U(2), the state of the final N-1 spins can provide *no information* about the similarity frame of spin #1.

This problem is already present for meronomic frame information. We can provide quantum resources for specifying how tictacs can be divided into qubits, but this protocol presumes that the decomposition of the world into tictacs is already given. That decomposition can be encoded into states of even larger systems, but at every stage we must presume the decomposition of a bigger universe into larger chunks. The meronomic frame problem gets worse as we introduce more quantum resources to resolve it.

## 6. Remarks

We have avoided giving a formal definition of the “interpretation” of a theory. However, informally, we might say that an interpretation is a set of rules for extracting meaning from the mathematical formalism of a theory. In quantum mechanics, the formalism includes a global quantum state that evolves unitarily. The many-worlds interpretation claims to extract from this formalism various meaningful statements about processes and correlations, including observations made by observer subsystems.

The problem is that any mathematical framework of states and time evolution maps (S and K) entails a group of automorphisms, which we have called “similarities”. These similarities may be time-independent, or they may be time-dependent (like the shift from *green/blue* color language to *grue-bleen* color language). When viewed through the spectacles of a similarity transformation, one particular instance of a theory is transformed into another. In some cases—including unitary quantum mechanics—*any* instance can be transformed into *any* other.

The complex universe Q of Section 1.2 seems very different from the simple universe Q’, and any interpretational approach that cannot distinguish them is plainly inadequate. However, the two universes are related by a similarity transformation of the underlying theory—they are, in effect, two pictures of the *same* universe. How is our interpretation to distinguish them? The only way to fix this problem is to impose a restriction on the set of similarities.

If we regard quantum theory as a pragmatic set of rules that an observer applies to analyze a limited, external system, then such a restriction is reasonable. It may arise, not from anything “inside” the system itself, but from the relationship between the observer and the system. The observer may well insist on this additional structure before applying the theory. However, the many-worlds program requires that we regard quantum theory as a description of an entire universe that includes the observer. Recall that Everett titled his detailed account “The Theory of the *Universal* Wave Function” ([2], emphasis ours).

We are left with a quandary. We must appeal to additional “frame” information beyond S and K in order to apply quantum theory in a meaningful way. This information is not quantum information—that is, information residing in the state of the system of interest. The interpretational frame is not a quantum reference frame. However, if we simply require this frame information on pragmatic grounds, as a mere prerequisite for applying the theory, we have forfeited one of the central motivations of the many-worlds interpretation. Inasmuch as the many-worlds program aims to implement DeWitt’s maxim—that the mathematical formalism of quantum mechanics can yield its own interpretation—that program fails.

The reader may wonder whether this is simply a new type of many-worlds situation. Perhaps every different possible “picture” of an evolving quantum system is equally meaningful, and a full interpretation embraces them all. However, this will not do. The “worlds” represented in a quantum state correspond to distinct branches or superposition components of the global quantum wave function. The different branches evolve independently according to a given time evolution U(t). This allows us to make conditional predictions, e.g., “Given that the observer’s record of the previous spin measurement is that $S_{z}=+\frac{\hbar }{2}$, the next measurement will yield the same result.” However, the many-pictures idea supports no sort of predictability at all. All possible time-evolutions, including those with wildly varying Hamiltonians H(t), are equally admissible pictures of the same universe. We cannot use the past behavior of the universe, or our present records of that behavior, to make any reliable prediction of future events. A many-pictures approach can yield no meaningful interpretation.

We have seen some simple theories (e.g., symbolic dynamics) in which non-trivial interpretational statements are possible even with maximal similarity groups U and $U^{\left(\mathrm{ext}\right)}$. On the other hand, the same difficulties do arise in classical Hamiltonian mechanics. This has not usually been recognized as a problem because the ordinary classical dynamical variables—for instance, the relative positions of particles in space—are generally assumed to have immediate physical meanings. Only with the introduction of quantum mechanics are interpretational issues recognized.

Obviously, we are able to use both classical and quantum mechanics to analyze the behavior of systems, extracting meaningful interpretational statements. We resolve the similarity problem, just as we resolve the *grue-bleen* color language problem, by appealing to objects and procedures that are not contained within the system of interest. In this view, we always interpret quantum mechanics by appealing, implicitly or explicitly, to sectors of the universe that are not treated as parts of the quantum system. In so doing, we presume that these external entities do not themselves have interpretational ambiguities. Their dynamical variables have immediate physical meaning; their reference frames for subsystem decomposition and time evolution are given. They provide our frame for interpreting the quantum physics of the system of interest. In addition, this is true even if we formally adopt a many-worlds view of the system and its behavior—or to put the same point another way, *a truly isolated quantum system has no interpretation.*

In this paper, we have not proposed or endorsed any particular interpretation of quantum mechanics. Many interpretations seem to offer valuable insights; none of them seem entirely satisfactory. Our point is simply that any successful interpretation—any interpretation that generates non-trivial interpretational statements about a theory—must somehow limit the similarity groups U and $U^{\left(\mathrm{ext}\right)}$ for that theory. However, the mere mathematical structure of Hilbert space and unitary operators does not appear to offer a way to do this. We are fully in agreement with Wallace’s cautionary remark about “additional structure”. Without a resolution of the quantum “grue-bleen” problem, no meaningful interpretation is possible.

The traditional “Copenhagen” interpretation of quantum mechanics relies on a conceptually independent macroscopic “classical” domain [16,17]. The interaction of subsystems becomes a measurement when the measurement record is irreversibly amplified into this domain. The quantum evolution of an isolated system has no meaning except that given by the possible results of such measurement processes. As John Wheeler said, “No elementary phenomenon is a phenomenon until it is an observed phenomenon” [18].

Thus, although we do not defend any particular interpretation, our considerations here lead us toward a Copenhagen-style point of view. In some theories, including quantum mechanics, we simply cannot construct a viable interpretation of a system based only on the states and dynamical evolution of the system itself. The physical basis for any interpretation must lie outside the system—not necessarily as a separate “classical” domain, but as a domain that is somehow excluded from the similarity transformations implicit in the mathematical formalism of the theory.

An analogy to our situation may perhaps be found in axiomatic set theory. Given any set *X*, a larger one can be found (e.g., by forming the power set P(X)). Thus, there is no upper limit to the size of the objects describable in the theory. However, the collection of all sets is not a self-consistent set. The “universe” of set theory is not an object within the theory [19].

Perhaps something similar holds for physical theories like quantum mechanics. There is no fundamental limit to the size of the system that can have a non-trivial interpretation. Even a large system could be embedded in a still larger system that provides the necessary interpretational frame. If we in turn wish to treat the larger system within the theory, we can (in principle) embed it in a simply enormous “super-system” to fix its frame. However, it is not possible to have a non-trivial interpretation for a quantum system that includes the entire universe.

The authors gratefully acknowledge many helpful comments from Chris Fuchs, Rob Spekkens, Bill Wootters, Austin Hulse, and Fred Strauch. They are of course not responsible for the remaining shortcomings of this paper.

## References

[1] Everett H. (1957). Relative State Formulation of Quantum Mechanics. Rev. Mod. Phys..

[2] Everett H., DeWitt B.S., Graham N. (1973). The Theory of the Universal Wave Function. The Many-Worlds Interpretation of Quantum Mechanics.

[3] Hartle J.B. (1968). Quantum Mechanics of Individual Systems. Am. J. Phys..

[4] Zurek W.H. (2005). Probabilities from Entanglement, Born’s rule *p_k_* = |*ψ_k_*|^2^ from Envariance. Phys. Rev. A.

[5] Zurek W.H. (1982). Environment-Induced Superselection Rules. Phys. Rev. D.

[6] Paz J.P., Zurek W.H., Kaiser R., Westbrook C., David F. (2000). Environment-induced decoherence and the transition from quantum to classical. Decoherence: Theoretical, Experimental, and Conceptual Problems.

[7] Joos E., Zeh H.D., Keifer C., Guilini D., Kupsch J., Stamatescu I.-O. (2003). Decoherence and the Appearance of a Classical World in Quantum Theory.

[8] DeWitt B.S. (1970). Quantum Mechanics and Reality. Phys. Today.

[9] Wheeler J.A. (1957). Assessment of Everet’s ‘Relative State’ Formulation of Quantum Theory. Rev. Mod. Phys..

[10] Graham N., DeWitt B.S., Graham N. (1973). The Measurement of Relative Frequency. The Many-Worlds Interpretation of Quantum Mechanics.

[11] Peres A. (1995). Quantum Theory: Concepts and Methods.

[12] Goodman N. (1955). Fact, Fiction, and Forecast.

[13] Wallace D. (2012). The Emergent Multiverse: Quantum Theory according to the Everett Interpretation.

[14] Hulse A., Schumacher B. (2019). Quantum meronomic frames. arXiv.

[15] Bartlett S.D., Rudolph T., Spekkens R.W. (2007). Reference frames, superselection rules, and quantum information. Rev. Mod. Phys..

[16] Bohr N. (1928). The quantum postulate and the recent development of atomic theory. Nature.

[17] Heisenberg W., Pauli W. (1955). The Development of the Interpretation of the Quantum Theory. Niels Bohr and the Development of Physics.

[18] Wheeler J.A. (1979). Frontiers of Time.

[19] Halmos P.R. (1960). Naive Set Theory.

